# Electrophysiological Correlates of the Threshold to Detection of Passive Motion: An Investigation in Professional Volleyball Athletes with and without Atrophy of the Infraspinatus Muscle

**DOI:** 10.1155/2013/634891

**Published:** 2013-01-14

**Authors:** José Inácio Salles, Victor Rodrigues Amaral Cossich, Marcus Vinicius Amaral, Martim T. Monteiro, Maurício Cagy, Geraldo Motta, Bruna Velasques, Roberto Piedade, Pedro Ribeiro

**Affiliations:** ^1^Neuromuscular Research Laboratory, National Institute of Traumatology and Orthopaedics (INTO), Avenida Brasil 500, 20940-070 Rio de Janeiro, RJ, Brazil; ^2^Brazilian Volleyball Confederation, Shopping Città America Avenida das Américas 700, Bloco 7, Barra da Tijuca, 22640-100 Rio de Janeiro, RJ, Brazil; ^3^Biomedical Engineering Program, Centre of Technology, Federal University of Rio de Janeiro, Avenida Horácio Macedo 2030, Bloco H, Sala 327, Cidade Universitária, 21941-901 Rio de Janeiro, RJ, Brazil; ^4^Brain Mapping and Sensorimotor Integration Laboratory, Institute of Psychiatry, Federal University of Rio de Janeiro, Avenida Venceslau Brás 71, Botafogo, 22290-140 Rio de Janeiro, RJ, Brazil; ^5^Institute of Applied Neuroscience (IAN), Rua Pacheco Leão 704, 25 Jardim Botânico, 22460-030 Rio de Janeiro, RJ, Brazil

## Abstract

The goal of the present study is to compare the electrophysiological correlates of the threshold to detection of passive motion (TTDPM) among three groups: healthy individuals (control group), professional volleyball athletes with atrophy of the infraspinatus muscle on the dominant side, and athletes with no shoulder pathologies. More specifically, the study aims at assessing the effects of infraspinatus muscle atrophy on the cortical representation of the TTDPM. A proprioception testing device (PTD) was used to measure the TTDPM. The device passively moved the shoulder and participants were instructed to respond as soon as movement was detected (TTDPM) by pressing a button switch. Response latency was established as the delay between the stimulus (movement) and the response (button press). Electroencephalographic (EEG) and electromyographic (EMG) activities were recorded simultaneously. An analysis of variance (ANOVA) and subsequent post hoc tests indicated a significant difference in latency between the group of athletes without the atrophy when compared both to the group of athletes with the atrophy and to the control group. Furthermore, distinct patterns of cortical activity were observed in the three experimental groups. The results suggest that systematically trained motor abilities, as well as the atrophy of the infraspinatus muscle, change the cortical representation of the different stages of proprioceptive information processing and, ultimately, the cortical representation of the TTDPM.

## 1. Introduction

The atrophy of the infraspinatus has been clinically recognized as corresponding to a suprascapular nerve palsy [1]. The suprascapular nerve is sensory and motor in nature, and it provides motor innervations to the infraspinatus and supraspinatus muscles [2, 3]. Atrophy of the infraspinatus muscle is an uncommon pathology, usually observed in professional athletes [4]. Inadequate training techniques and premature specialization contribute to peripheral neurological lesions of the athletes' shoulders [5]. Suprascapular nerve injury is usually incomplete, allowing asymptomatic sport performance, because of the compensatory action of teres minor [6]. Holzgraefe et al. [7] showed that 33% of high level volleyball players had clinical or electrophysiological evidence of suprascapular nerve injury. More recently, Alary et al. [8] identified a 30% incidence of infraspinatus atrophy in beach volleyball athletes.

The high incidence of this pathology in volleyball players suggests that the nature of the game plays an important role in the pathogenesis of the atrophy of the infraspinatus muscle [9]. The pathogenesis of this injury lies in the floating service, though the authors admit the possibility that some players might be susceptible to the lesion due to a predisposition caused by anomalous factors of the terminal branch of the nerve, hypertrophy of the spinoglenoid ligament or the high mobility of the shoulder, among others [10, 11]. We hypothesized that athletes with infraspinatus muscle atrophy can develop proprioceptive deficits, which might be explained by the fact that mechanoreceptors of the posterior capsule and glenohumeral joint are mechanically sensitive and transduce mechanical tissue deformation as frequency-modulated [10, 12–14]. Proprioception is a specialized form of the sense that encompasses the ability to detect movement [15]. The assessment of proprioception is determined by the ability to detect joint movement and has been traditionally conducted by measuring the threshold to detection of passive motion (TTDPM) [7, 14, 16]. 

Proprioceptive ability in the shoulder is essential for correctly positioning the hand during serving and spiking in a volleyball match. In our knowledge, no investigations were published about proprioceptive deficits in a group of individuals with suprascapular neuropathy and no studies were conducted about the pattern of these deficits in the central nervous system. The identification of suprascapular neuropathy in elite volleyball players has suggested that a combination of traction, friction, and kinking of the nerve at points of tethering may induce nerve injury [17]. This may be particularly true at the spinoglenoid notch, a site which anatomic studies have demonstrated an increase in spinoglenoid ligament tension against the nerve in the positions that correspond to the follow-through phase of throwing [18, 19]. Combined scapular protraction and infraspinatus contraction during this phase may further bowstring the nerve against the scapular spine, with acute and/or chronic injury resulting [20]. There is a possibility of the passive motion detection during internal rotation of the shoulder activating the mechanoreceptors of the posterior capsule primarily in relation to receptors located in the ligaments and labrum. This suggests that the athletes with atrophy of the infraspinatus, with possible involvement of the suprascapular nerve, may show a delay in the detection of passive motion when compared to their peers without muscle atrophy. Consequently, such delay might be reflected in the cortical representation of stimulus processing.

In this study, we hypothesized that infraspinatus muscle atrophy secondary to suprascapular nerve injury could provide proprioception deficit in shoulder joint. The proprioceptive deficits can be defined through the measure of TTDPM [15, 16]. The TTDPM is a tool to quantify one's ability to consciously detect joint movement. The use of cortical representation of passive motion associate to the TPPM is a electrophysiological measure and is a new paradigm to access the brain activation related to proprioception [21–23]. In literature, there is just description of use of cortical representation with disable individuals and healthy controls subjects, and not mention professional athletes [21–23]. In this sense, we believe it is important to apply this kind of evaluation in professional athletes who presents an anatomical disorder in the shoulder joint, trying to understand the patterns of neural activity related to somatosensory perception [24, 25]. We also hypothesized that the different frequency bands (i.e., alpha and beta) are able to discriminate the unbalance provoked by the infraspinatus muscle atrophy. Specifically, we anticipated that frontal and central regions are able to discriminate the three different groups in relation to the cognitive and motor aspects. On the other hand, we hypothesized that parietal cortex is able to differentiate the sensory component of the passive movement. Once these hypotheses could be proved, it will be possible to develop specific exercises programs to rehab the sporting act, to minimize the consequences of the orthopedic disorders to professional athletes, allowing them to improve their performance.

Electrophysiology has also been employed in the study of proprioception. Specifically, electrophysiological studies have investigated the cortical representation of passive motion [8, 21–23]. In this context, the goal of the present study is to compare the electrophysiological variables, that is, alpha and beta frequency, of the TTDPM among a group of healthy individuals (control group), a group of professional volleyball athletes with atrophy of the infraspinatus muscle on the dominant side, and a group of athletes with no shoulder pathologies. More specifically, the study aims at assessing the effects of infraspinatus muscle atrophy on the cortical representation of the TTDPM. 

## 2. Methodology

### 2.1. Sample

**Figure 1:**
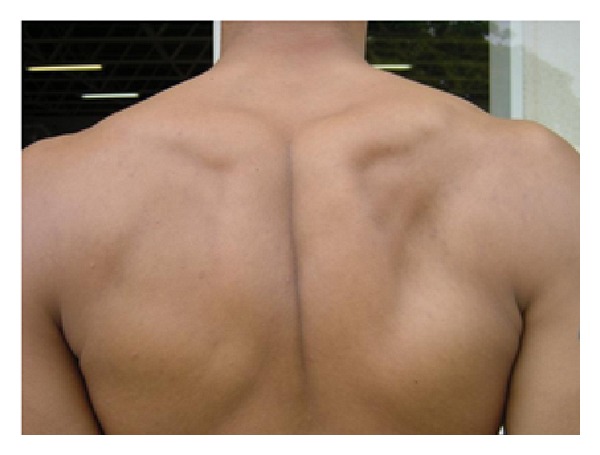
Professional volleyball player with atrophy of the infraspinatus muscle on the dominant side.

The sample of the study consisted of 58 right-handed male volunteers, with ages ranging from 18 to 26 years old: 18 professional volleyball players with atrophy (PAG) of the infraspinatus muscle on the dominant side (Figure 1), 20 volleyball players without the atrophy (PG) of infraspinatus, and 20 healthy nonathlete controls (CG) (Table 1). All athletes have an international high level of competition, with a mean of 12 years of experiences playing volleyball. They were in perfect clinical condition to engage in physical activities. Control subjects had no previous history of shoulder pathologies on the dominant side and did not participate in any systematic long-term activities to improve upper limb abilities. All participants were healthy, free of cognitive deficits and were not using any medication or psychoactive substance at the time of the test. The diagnostic of the infraspinatus atrophy was confirmed by an orthopedic surgeon after clinical exam. The definition of infraspinatus atrophy was loss of muscle belly in the infraspinatus scapular fossa in a posterior shoulder view. The atrophy was considered present or absent, without quantifying the level of infraspinatus atrophy, once this is a controversial topic, because it could be influenced by factors as genetic, exercises and nutrition. The Edinburgh Handedness Inventory was used to assess laterality and exclude left-handed individuals from the experiment. Prior to their inclusion in the study, participants signed a consent form, where the experimental conditions were thoroughly described. The ethics committee of the Psychiatric Institute of the Federal University of Rio de Janeiro approved the experiment. 

### 2.2. Apparatus

**Figure 2:**
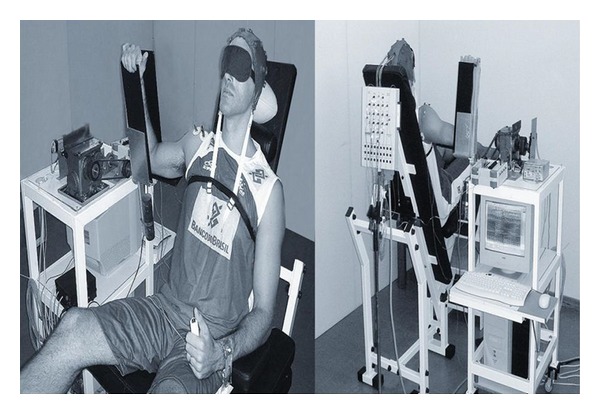
The apparatus and participant position for the experiment procedure.

A motor-driven, proprioception testing device (PTD), which was developed at the Neuromuscular Research Laboratory of the National Institute of Traumatology and Orthopaedics (INTO) [26], was used to assess the TTDPM (Figure 2). The PTD comprised (1) a motor reducer driver that included an electric motor (12 V, 12 Watts, 7 A, 14 Nm torque) and a reducer; (2) synchronized pulleys and a belt that moved a lever arm; (3) a u-shaped lever arm for limb placement; (4) air splints around the lever arm and the participant's arm to provide uniform compression within the device, stabilize the upper extremity, and reduce cues from cutaneous mechanoreceptors [27]; (5) a button switch for the participant to indicate a response. The device was designed to passively move the arm in internal and external rotations of the shoulder joint. A potentiometer connected to the shaft of the lever arm and interfaced with a PC converted angular movement into electric signals that were stored in the computer. 

Electroencephalographic (EEG) activity was acquired during the task with a 20-channel Braintech-3000 (EMSA-Medical Instruments, Rio de Janeiro, Brazil). The International 10/20 System [28] for electrode placement (referenced to linked earlobes) was used and the 20 monopolar electrodes were arranged in a nylon cap. Electromyographic (EMG) activity of the deltoids and pectoralis major muscles [15, 29] was recorded concurrently with the EEG by an EMG1000 device (Lynx, São Paulo, Brazil).

### 2.3. Task and Procedure

A light- and sound-attenuated room was prepared for data acquisition. Participants were comfortably seated on a reclining chair attached to the PTD, with the dominant shoulder abducted at 90 degrees and rotated forward by 30 degrees so that the arm was in the same plane as the scapula. The elbow was flexed at 90 degrees [30, 31]. Participants were instructed to relax and to avoid imagining movement of the shoulder during the task. The task involved internal rotations of the shoulder around its longitudinal axis, from the initial position established at 80 degrees of external rotation. This position was chosen to avoid extreme positioning of the joint. Auditory and visual cues were attenuated using ear-plugs and a blindfold. Each trial started with 15s of EEG and EMG recording without motor movement. After these baseline measurements, the motor shaft was engaged to rotate the shoulder at the constant rate of 0.4 degrees/s in the direction of the internal rotation. Participants were instructed to respond by pressing the hand-held switch when movement of the shoulder was detected. Participants performed five blocks of four trials each. TTDPM was measured by recording the response latency, that is, the time to detect the passive movement and press the button switch after stimulation onset.

### 2.4. EEG and EMG Data Acquisition

The software Data Acquisition developed at Neuromuscular Research Laboratory (INTO, Rio de Janeiro, Brazil) used to acquire the EEG signal was developed at the. Visual inspection was used to detect and eliminate artifacts in the recordings. The data acquired had total amplitude of less than 100 *μ*V. The EEG signal was amplified with a gain of 22.000, analogically filtered between 0.1 Hz (high-pass) and 50 Hz (low-pass) [32] and digitalized with a sampling rate of 200 Hz. A digital 60 Hz notch filter was employed. Eye-movement (EOG) artifact was monitored with two bipolar electrodes (9 mm diameter) attached above and on the external canthus of the right eye. Impedance for EEG and EOG electrodes was under 5 kΩ and 20 kΩ, respectively. EMG activity was sampled at 1,000 Hz.

### 2.5. Data Processing

Data collected during the experiment were processed with Matlab 5.3 (MathWorks, M, USA). The data were first averaged for each participant and then across participants. Considering all passive movement repetitions, response latency (i.e., millisecond) was established as the delay between the onset of the stimulus (passive movement) and the response (button press). EEG epochs were aligned to the pressing of the button-switch (trigger). The presence of EMG activity was used as an exclusion criterion, only to detect voluntary movement, since it signalizes the volunteer did not remain relaxed. Root mean square (RMS), defined as the square root of the mean square value for the EMG was calculated [29, 33] and used to assess possible voluntary movement to the motion by the participant. Continuous EEG data were epoched in 9000-millisecond windows time-locked to the trigger. The baseline was set between −2000 ms and 0, similarly to previous works from our group [34–36], the analysis period of interest between 0 and 7000 ms, in order to more information about the movement perception and detection. The data were submitted to Independent Component Analysis (ICA), which was implemented in EEGLAB [8] under the Matlab platform, in order to remove the components that eminently contained artifacts. 

### 2.6. Statistical Analysis

Response latencies and absolute power at beta and alpha frequencies were the dependent variables of interest. Statistical analysis was performed using SPSS for Windows—version 17.0 (SPSS Inc., Chicago, USA) and we employed an ANOVA one-way with repeated measure with subsequent post hoc tests to assess possible differences among the experimental groups for response latencies. To assess cortical activity, we analyzed absolute power at beta and alpha frequencies on the electrodes F3, C3 and P3. We performed an ANOVA two-way with repeated measure to investigate the relationship between the factor group (3 levels: professional volleyball players with atrophy of the infraspinatus muscle on the dominant side; volleyball players without the atrophy of infraspinatus; and healthy nonathlete controls) and the factor moment (2 levels: moment 1 represents 2 s before pressing the button switch; moment 2 represents 2 s after pressing the button switch). The group differences were tested using Scheffè post hoc test if ANOVA was significant.

## 3. Results

### 3.1. Latency

**Figure 3:**
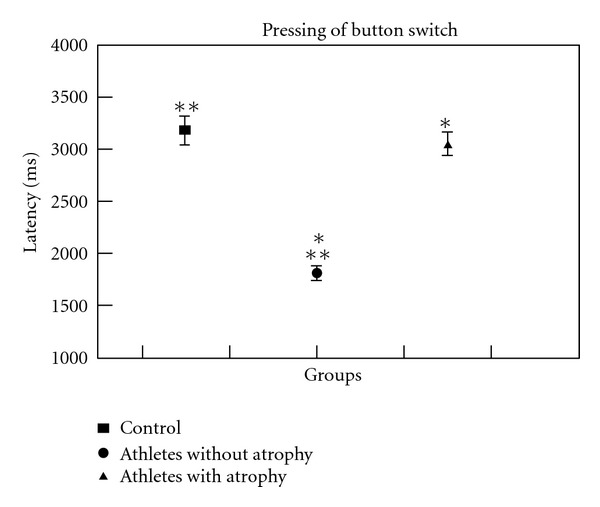
Latency variations among groups. The statistical analysis revealed that PG differs from PAG and CG (*P* < 0.001).

The behavioral measure demonstrated a statistical difference between the PG when compared both to the PAG and to the CG (Table 2). Specifically, the PG was significantly faster than the PAG (*P* < 0.001) and faster than the CG (*P* < 0.001). The PAG did not differ from the CG (*P* = 0.861) (Figure 3).

### 3.2. Quantitative Electroencephalography

**Figure 4:**
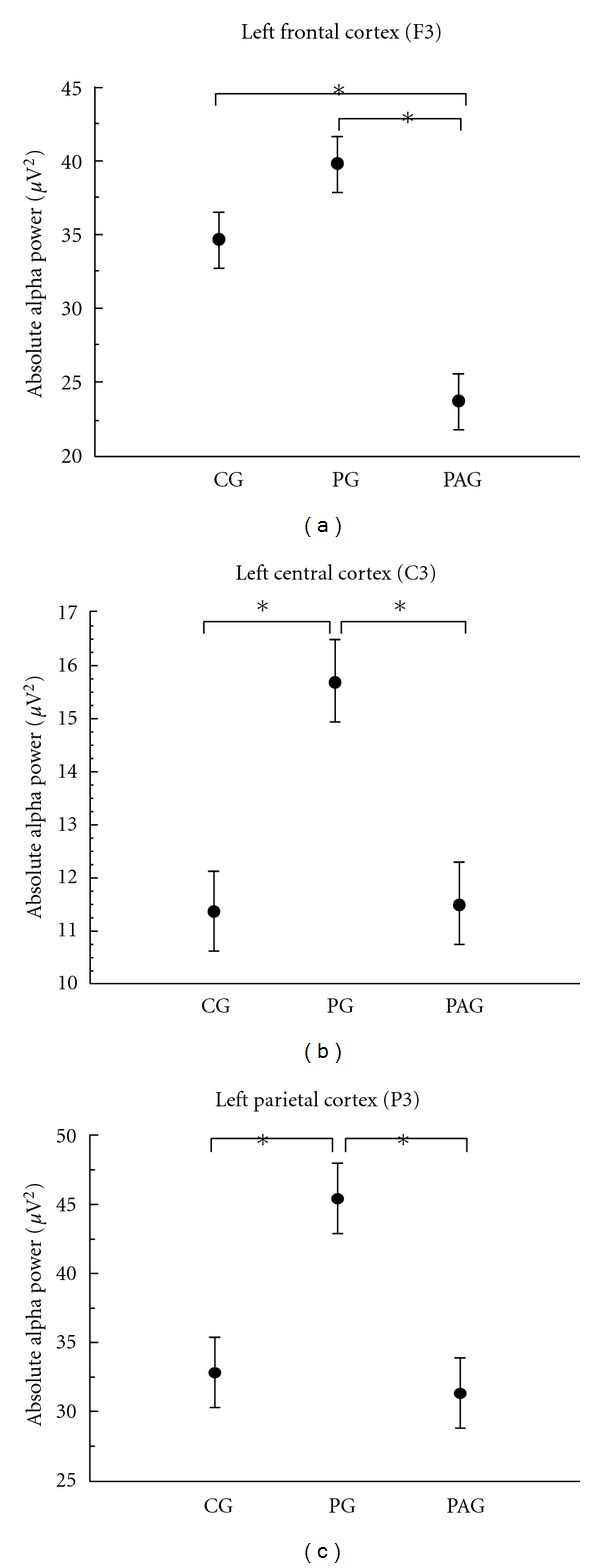
Mean and standard deviation of absolute alpha power. The statistical analysis revealed a main effect for group (*P* < 0.001) for all the electrodes analyzed. (a) Left frontal cortex (F3). (b) Left central cortex (C3). (c) Left parietal cortex (P3).

Alpha Frequency. For the left frontal cortex (F3), we found a main effect for group with an absolute alpha power increase for the PG and an absolute alpha power decrease for the PAG (*F* = 19,050, *P* < 0.001, observed power = 0.998) (Table 2). The post hoc analysis demonstrated that for F3 electrode the PAG differs from the PG and the CG (Figure 4(a)). For the left motor cortex (C3) (*F* = 10,380; *P* < 0.001, observed power = 0.988) (Figure 4(b)) and the left parietal cortex (P3) (*F* = 9,181; *P* < 0.001, observed power = 0.976), we observed a main effect for group with an absolute alpha power increase for the PG, and the post hoc analysis revealed that the PG is different from the CG and the PAG (Figure 4(c)).

**Figure 5:**
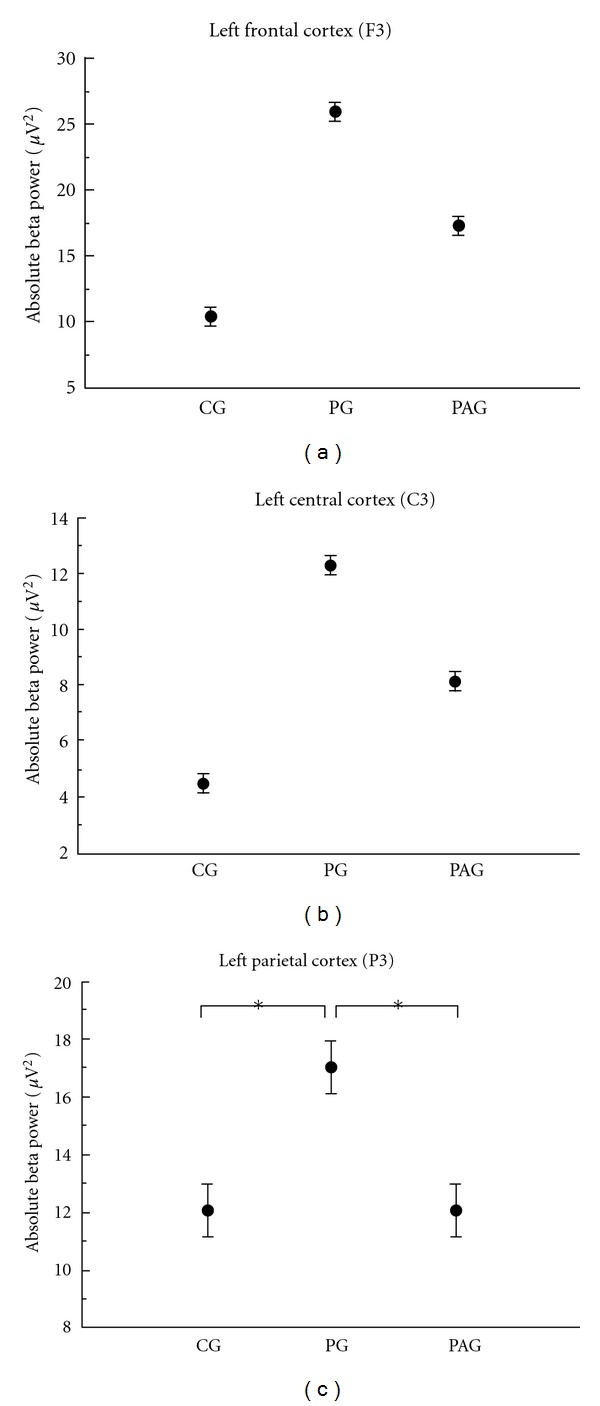
Mean and standard deviation of absolute beta power. The statistical analysis revealed a main effect for group (*P* < 0.001) for all the electrodes analyzed. (a) Left frontal cortex (F3). (b) Left central cortex (C3). (c) Left parietal cortex (P3).

Beta Frequency. We found a main effect for group in the three electrodes investigated, that is, F3 (*F* = 117,144; *P* < 0.001, observed power = 0.968), C3 (*F* = 130,884; *P* < 0.001, observed power = 0.982), and P3 (*F* = 11,096; *P* < 0.001, observed power = 0.992) (Table 2). For the left frontal cortex (F3) (Figure 5(a)) and the left central cortex (C3) (Figure 5(b)), the groups are different among them; we observed a higher absolute beta power for the PG and a lower absolute beta power for the CG. For the left parietal cortex (P3), we found the same pattern; however, the CG and the PAG did not differ between them (Figure 5(c)).

## 4. Discussion

The goal of the present study is to assess proprioception through the characterization of the TTDPM during internal rotation of the shoulder in professional volleyball athletes with atrophy of the infraspinatus muscle, and to assess the effects of the atrophy on the cortical representation of the TTDPM. We hypothesized that (1) athletes with atrophy of the infraspinatus show a greater delay in the detection of passive motion when compared to their peers without muscle atrophy. Pap et al. [37] identified that patient with anterior cruciate ligament- (ACL-) deficient knees failure to detect movement when compared with healthy subjects [37, 38]; (2) different frequency bands (i.e., alpha and beta) are able to discriminate the unbalance provoked by the infraspinatus muscle atrophy [39]; (3) the pattern of cortical activity in the PAG would differ from the PG and the CG, as a consequence of a proprioceptive deficit [37]. Correspondingly, we expected to see the expression of the proprioceptive deficit as an increase in the latency measure of the TTDPM. In other words, we expect the PAG to show a greater delay than the CG and the PG due to a possible proprioceptive deficit caused by the atrophy. Our results support our hypothesis, and we will discuss them below.

### 4.1. Latency

Researches have been conducted in the attempt to study proprioceptive deficits as assessed by the TTDPM. Lephart et al. [40] examined the TTDPM in normal subjects and found no significant differences between their dominant and nondominant shoulders. Other studies have demonstrated proprioceptive deficits in shoulders with glenohumeral dislocation [30] and in the dominant shoulder of healthy overhead athletes when compared to their nondominant shoulder [3]. Von Drongele [32] has identified Pacinian corpuscles in glenohumeral ligaments, Ruffini-like ending in the glenohumeral capsule, and the free nerve endings in the glenoid labrum of human cadavers. The changing views of the role of the joint receptors in kinesthetic are well described in Matthews [41]. It is believed that passive proprioception assessment predominantly measures the stimulation of joint mechanoreceptors during movement sense by minimizing muscle receptors involvement [42]. In our study, we used the LTPM. The shoulder of the subjects was passively moved from the starting position and the RMS of the EMG was used as an exclusion criterion when the muscle contraction was identified. 

The present study, therefore, substantiates these previous findings by showing that the PAG also presents proprioceptive deficits as assessed by the TTDPM. The PAG showed a significant delay in motion detection when compared to the PG and the CG. Moreover, PAG athletes have a behavior response similar to the CG. The two athlete groups were not different, a priori, with respect to two factors: training level and technical expertise. However, the two groups of athletes differed with respect to the presence of atrophy of the infraspinatus. The results indicate that there are significant differences in latency. Specifically, the PG detected the stimulus (i.e., passive motion) faster when compared to the PAG. This result supports the view that atrophy of the infraspinatus is a limiting factor for athletes, increasing the time of response to a sensory stimulus and, in this case, of passive motion detection.

The results indicate a significant difference between the two groups, the PG responded significantly faster, confirming that level of training and technical expertise are key factors in reducing the latency of detection of passive motion. Finally, when the PAG and the CG are compared, the results show no significant differences between groups. This result suggests that atrophy of the infraspinatus is a significant factor in the TTDPM increase and in the consequent speed reduction of the stimulus processing. 

### 4.2. Alpha and Beta Frequencies

Our electrophysiological results showed a greater absolute beta and alpha power for the PG on the three electrodes investigated (i.e., C3, F3 and P3). The C3 and F3 electrodes represent the primary motor cortex and the premotor cortex, respectively. They are part of the sensorimotor cortex and are responsible for the movement planning and control [43, 44]. On the other hand, P3 electrode represent the posterior parietal lobe, area that plays an important role in integrating sensory information from various parts of the body [35]. Regarding absolute alpha power, we identified that the PAG demonstrated a power decrease when compared with the CG and PG for the F3 electrode. However, for C3 and P3 electrodes we did not find difference between the CG and PAG. When we investigate beta power, we found a difference among the three groups for the electrodes representing the sensorimotor areas (i.e., F3, and C3); for the P3 electrode, we did not find difference between the CG and PAG.

Beta frequency is more sensible to sequencial and repetitive movement than alpha frequency [45, 46]. In a more general sense, beta is associated with movement preparation and contralateral movement [21, 47] while evidence indicates that alpha frequency oscillations may be a correlate of activated cortical areas during sensory and motor processing [48]. Müller et al. [39] investigated beta electroencephalographic (EEG) changes during active and passive hand movements. They found that sensorimotor processing during passive hand movements involves some of the processes which are also involved in voluntary hand movements. Our results are in agreement with this, and we found that beta power is more sensible than alpha power in terms to discriminate and detect movement, since we verified that F3 and C3 beta power are different among the three groups. In other words, absolute beta power is able to discriminate the unbalance provoked by the infraspinatus muscle atrophy, mainly when we investigate areas related to movement detection. We found that the PAG shows a different pattern of cortical activity during the passive movement task when compared to the PG, demonstrating that the infraspinatus muscle atrophy provoke changes in the cortical representation, as supported by Müller et al. [39] that demonstrated a similar process in active and passive movement. The two experimental groups (i.e., PAG and PG) have the same training level and technical expertise, and they only differed with respect to the presence of atrophy of the infraspinatus. Then, the representation of cortical activity in the PAG would differ from the PG, as a consequence of a proprioceptive deficit [37].

The PG shows a high frequency oscillation concentrated around the motor response, suggesting that this group needed less time to detect the stimulus (i.e., passive motion) and started to prepare for the motor response (pressing the button switch) earlier in time. These data interpretations are supported by the latency results mentioned above, which showed that the PG was significantly faster in detecting passive motion when compared to the other two groups. In the CG, on the other hand, the oscillations pattern was spread and sustained in time, suggesting that this group needed more time to detect the stimulus, process the sensory information, and respond. 

In the frontal (F3) and central (C3) regions, we found difference among the three groups (CG, PG, and PAG). The frontal region (F3) close related to the premotor cortex and the central area (C3) associated primary motor cortex were able to discriminate among the three groups. Specifically, these two regions which are associated directly with motor aspects could detect the difference between PG and PAG. Differently, the parietal region which traditionally is responsible to integrate different sources of information was not able to detect the difference between PG and PAG. The left parietal cortex in the absence of sensory information associated with infraspinatus muscle atrophy considered CG equals to PAG. Considering all the results together, beta absolute power supports our hypothesis on the sense that we can correlate different aspects of the pathology (e.g., infraspinatus muscle atrophy) with electrophysiological changes.

Overall, the results suggest that both consistently trained motor skills and the atrophy of the infraspinatus muscle alter the cortical representation of the various stages of proprioceptive information processing and, ultimately, the TTDPM cortical representation. In sum, by taking into account the different analyses of this study, we suggest that the dynamics of proprioceptive processing between the PAG and the PG is quantitatively different, as reflected by the significant difference in latency, and alpha and beta frequencies values. Future studies are necessary to thoroughly understand the effects of infraspinatus muscle atrophy as well as other pathologies on the proprioceptive abilities of athletes. Understanding the effects of this and other pathologies is fundamental for more adaptive training and treatment strategies of professional athletes. Future studies are also necessary to further explore the issue of the cortical representation of sensory deficits in different populations.

Our study has limitations related to clinical evaluation of infraspinatus atrophy and how to explain the pathophysiology of this condition. In the methodology proposed, we just define the presence of infraspinatus atrophy, once this clinical find cannot be quantified by physical exam, so we just did a qualitative analysis. We believe to quantify this atrophy is necessary the use of images of magnetic resonance, so that it could be graduate. Certainly it would be part of other study. Other issue is related to the pathophysiology of this isolated infraspinatus muscle atrophy. The literature states that isolated infraspinatus atrophy suggests selective suprascapular nerve injuries, specifically at the spinoglenoid notch, in the infraspinatus branch [49]. The main explanation for this type of injury is traction, or mechanical stretching of the nerve, with or without friction at spinoglenoid notch. This type of injury is exacerbated by simultaneous scapular protraction and infraspinatus contraction [6]. These traction forces and ranges may be accentuated by the extreme torque and angular velocity placed on the shoulder during overhead activities, as in the volleyball play [6]. In this scenario, the infraspinatus is the only muscle involved, and because of the incomplete nature of the lesion, there remains some function of the muscle, which allows for asympomatic activity. This may occur as a result of teres minor compensation for the infraspinatus dysfunction, what could be potentialized by a specific exercise program [49]. For future studies, we suggest the investigation of the TTDPM could be expanded to the contralateral shoulder and within-subject comparisons in addition to between-subject comparisons in order to observe if the TTDPM and the cortical representation differ between limbs.

## Figures and Tables

**Table 1:** Demographic table comparing the three groups.

	Volleyball players (*N* = 38) mean (SD)		Control group (CG) (*n* = 20) mean (SD)
	Athletes with atrophy (PAG) (*N* = 18)	Athletes without atrophy (PG) (*N* = 20)	
Age	29.4 (4.7)	32.5 (4.1)	30.3 (4.3)
Weight (Kg)	92.1 (8.3)	95.9 (9.2)	76.2 (9.2)
Height (cm)	196.0 (6.8)	198.4 (7.8)	181.2 (6.7)

**Table 2:** Mean and standard deviation of the variables dependents.

	Volleyball players (*N* = 38) mean (SD)		Control group (CG) (*n* = 20) mean (SD)
	Athletes with atrophy (PAG) (*N* = 18)	Athletes without atrophy (PG) (*N* = 20)	
			
Latency (ms)	4,180 (260)	2,237 (260)	4,202 (247)
Alpha-frequency (*μ*V^2^)			
F3	23.78 (1.924)	39.77 (1.785)	34.66 (1.879)
C3	11.51 (0.802)	15.68 (0.744)	11.36 (0.783)
P3	31.34 (2.679)	45.42 (2.486)	32.92 (2.617)
Beta-frequency (*μ*V^2^)			
F3	17.17 (0.753)	25.84 (0.699)	10.38 (0.736)
C3	8.12 (0.358)	12.28 (0.332)	4.50 (0.349)
P3	11.98 (0.91)	16.99 (0.844)	12.05 (0.888)
